# Correlation between acoustic rhinometry, computed rhinomanometry and cone-beam computed tomography in mouth breathers with transverse maxillary deficiency^☆^

**DOI:** 10.1016/j.bjorl.2016.10.015

**Published:** 2016-11-25

**Authors:** Raquel Harumi Uejima Satto Sakai, Fernando Augusto Lima Marson, Emerson Taro Inoue Sakuma, José Dirceu Ribeiro, Eulália Sakano

**Affiliations:** a Universidade Estadual de Campinas (Unicamp), Faculdade de Ciências Médicas, Departamento de Pediatria, Campinas, SP, Brazil; b Universidade Estadual de Campinas (Unicamp), Faculdade de Ciências Médicas, Departamento de Genética Médica, Campinas, SP, Brazil; c Universidade Estadual de Campinas (Unicamp), Faculdade de Ciências Médicas, Departamento de Radiologia, Campinas, SP, Brazil; d Universidade Estadual de Campinas (Unicamp), Faculdade de Ciências Médicas, Departamento de Oftalmologia e Otorrinolaringologia, Campinas, SP, Brazil

**Keywords:** Minimum cross-sectional areas, Nasal cavity, Respiratory flow, Average nasal resistance, Áreas mínimas de corte transversal, Cavidade nasal, Fluxo respiratório, Resistência nasal média

## Abstract

**Introduction:**

To provide clinical information and diagnosis in mouth breathers with transverse maxillary deficiency with posterior crossbite, numerous exams can be performed; however, the correlation among these exams remains unclear.

**Objective:**

To evaluate the correlation between acoustic rhinometry, computed rhinomanometry, and cone-beam computed tomography in mouth breathers with transverse maxillary deficiency.

**Methods:**

A cross-sectional study was conducted in 30 mouth breathers with transverse maxillary deficiency (7–13 y.o.) patients with posterior crossbite. The examinations assessed: (i) acoustic rhinometry: nasal volumes (0–5 cm and 2–5 cm) and minimum cross-sectional areas 1 and 2 of nasal cavity; (ii) computed rhinomanometry: flow and average inspiratory and expiratory resistance; (iii) cone-beam computed tomography: coronal section on the head of inferior turbinate (Widths 1 and 2), middle turbinate (Widths 3 and 4) and maxilla levels (Width 5). Acoustic rhinometry and computed rhinomanometry were evaluated before and after administration of vasoconstrictor. Results were compared by Spearman's correlation and Mann–Whitney tests (*α* = 0.05).

**Results:**

Positive correlations were observed between: (i) flow evaluated before administration of vasoconstrictor and Width 4 (Rho = 0.380) and Width 5 (Rho = 0.371); (ii) Width 2 and minimum cross-sectional areas 1 evaluated before administration of vasoconstrictor (Rho = 0.380); (iii) flow evaluated before administration of vasoconstrictor and nasal volumes of 0–5 cm (Rho = 0.421), nasal volumes of 2–5 cm (Rho = 0.393) and minimum cross-sectional areas 1 (Rho = 0.375); (iv) Width 4 and nasal volumes of 0–5 cm evaluated before administration of vasoconstrictor (Rho = 0.376), nasal volumes of 2–5 cm evaluated before administration of vasoconstrictor (Rho = 0.376), minimum cross-sectional areas 1 evaluated before administration of vasoconstrictor (Rho = 0.410) and minimum cross-sectional areas 1 after administration of vasoconstrictor (Rho = 0.426); (v) Width 5 and Width 1 (Rho = 0.542), Width 2 (Rho = 0.411), and Width 4 (Rho = 0.429). Negative correlations were observed between: (i) Width 4 and average inspiratory resistance (Rho = −0.385); (ii) average inspiratory resistance evaluated before administration of vasoconstrictor and nasal volumes of 0–5 cm (Rho = −0.382), and average expiratory resistance evaluated before administration of vasoconstrictor and minimum cross-sectional areas 1 (Rho = −0.362).

**Conclusion:**

There were correlations between acoustic rhinometry, computed rhinomanometry, and cone-beam computed tomography in mouth breathers with transverse maxillary deficiency.

## Resumo

**Introdução:**

Numerosos exames podem ser realizados para fornecer informações clínicas e diagnósticas em respiradores bucais com atresia maxilar e mordida cruzada posterior, entretanto a correlação entre esses exames ainda é incerta.

**Objetivo:**

Avaliar a correlação entre a rinometria acústica, rinomanometria computadorizada e tomografia computadorizada por feixe cônico em respiradores bucais com atresia maxilar.

**Método:**

Um estudo de corte transversal foi realizado em 30 respiradores bucais com atresia maxilar (7-13 anos) com mordida cruzada posterior. Os exames avaliados foram: (i) Rinomanometria Acústica: volumes nasais (0-5 cm e 2-5 cm) e áreas mínimas de corte transversal 1 e 2 da cavidade nasal; (ii) rinomanometria computadorizada: fluxo e nasal e resistências médias inspiratórias e expiratórias; (iii) tomografia computadorizada por feixe cônico: corte coronal na cabeça da concha inferior (larguras 1 e 2), concha média (larguras 3 e 4) e na maxila (largura 5). Rinomanometria Acústica e Rinomanometria Computadorizada foram avaliadas antes e depois da administração de vasoconstritor. Os resultados foram comparados pelo teste de correlação de Spearman e pelo teste de Mann-Whitney (α = 0,05).

**Resultados:**

Foram encontradas correlações positivas entre: (i) fluxo antes da administração de vasoconstritor e largura 4 (Rho = 0,380) e largura 5 (Rho = 0,371); (ii) largura 2 e área mínima de corte transversal 1 antes da administração de vasoconstritor (Rho = 0,380); (iii) fluxo antes da administração de vasoconstritor e volumes nasais de 0-5 cm (Rho = 0,421), 2-5 cm (Rho = 0,393) e área mínima de corte transversal 1 (Rho = 0,375); (iv) largura 4 e volume nasal de 0-5 cm antes da administração do vasoconstritor (Rho = 0,376), volume nasal de 2-5 cm antes do uso de vasoconstritor (Rho = 0,376), áreas mínimas de corte transversal 1 antes da administração de vasoconstritor (Rho = 0,410) e áreas mínimas de corte transversal 1 após o uso do vasoconstritor (Rho = 0,426); (v) largura 5 e largura 1 (Rho = 0,542), largura 2 (Rho = 0,411) e largura 4 (Rho = 0,429). Foram encontradas correlações negativas entre: (i) largura 4 e resistência inspiratória média (Rho = −0,385); (ii) resistência inspiratória média antes da administração de vasoconstritor e volume de 0-5 cm (Rho = −0,382), e resistência expiratória média antes da administração de vasoconstritor e área mínima de corte transversal 1 (Rho = −0,362).

**Conclusão:**

Correlações foram encontradas entre a rinometria acústica, a rinomanometria computadorizada e a tomografia computadorizada de feixe cônico em respiradores bucais com atresia maxilar.

## Introduction

Transverse maxillary deficiency can determine the presence of unilateral or bilateral posterior dental crossbite.^1^ Maxillary atresia with high-arched palate is one of the most frequent craniofacial bone alterations among mouth breathing children,^2^ and it may contribute to increased nasal airflow resistance due to the narrowing of the nasal cavity.^3^

The influence of mouth breathing on maxillary and dentofacial development is not clear in the medical literature.^4^ Current scientific reports are contradictory and basically embrace three different positions: (i) mouth breathers show a pattern of higher vertical growth than nasal breathers^5^; (ii) breathing patterns promote dental changes, but no facial changes^6^; (iii) mouth breathing does not influence craniofacial development.^7^, ^8^ One of the most common orthodontic procedures for the correction of transverse maxillary deficiency includes rapid maxillary expansion, whose effects to reduce mouth breathing pattern on a long-term basis still remain controversial.^9^, ^10^, ^11^, ^12^

Patients were evaluated with acoustic rhinometry (AR) and computed rhinomanometry (CR), which measure nasal respiratory function, as well as with cone-beam computed tomography (CBCT) of nasal cavity and maxilla, which evaluates the structure of the bone. CBCT images provide three-dimensional information for the evaluation of bone structures with less primary and secondary radiation than conventional radiographic tomography. Therefore, CBCT has been increasingly used by physicians and odontologists.^13^, ^14^, ^15^, ^16^

Recent studies have found correlation between volumes and nasal widths measures on computed tomography with nasal flow values of CR, and due to the reliability of AR, it has been considered along with the CT scan, as a reference method.^17^, ^18^

In this context, this study investigated whether a correlation between nasal respiratory function (AR and CR) and CBCT in mouth breathers with transverse maxillary deficiency exists.

## Methods

A total of 30 patients [12/30 (40%) males] between seven and thirteen years of age were included in this study. They were mouth breathers with transverse maxillary deficiency and unilateral or bilateral posterior crossbite, with no history of previous orthodontic treatment. Exclusion criteria included patients with extensive caries and syndromes.

**Figure 1:**
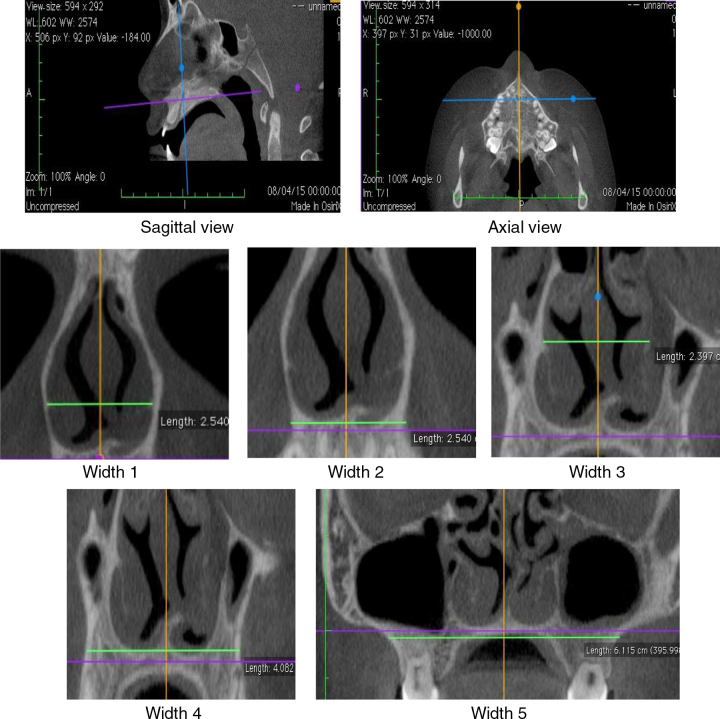
Sagittal and axial planes used for protocol for the reformatted coronal planes (Widths 1–5). Widths 1 and 2 – head of the inferior turbinate; Widths 3 and 4 – head of the middle turbinate; Width 5 – maxillary bone.

The diagnosis of mouth breathing was made by an otorhinolaryngologist or a physician at the Outpatient Clinic/Department of Otorhinolaryngology after clinical and nasofibroscopic examination.^19^, ^20^ In AR, measurements of minimum cross-sectional areas (MCA1 and MCA2) and volume (0–5 cm and 2–5 cm) of the nasal cavity were evaluated. CR assessed the measurements of nasal flow as well as the average inspiratory (AIR) and expiratory (AER) resistance before [without vasoconstrictor (WVC)] and after the use of vasoconstrictor. CBCT of maxilla and nasal cavity evaluated the widths of coronal sections at the levels of the head of the inferior turbinate (Widths 1 and 2), middle turbinate (Widths 3 and 4) and the maxilla (Width 5) (Fig. 1). Korkhaus analysis was performed on models of orthodontic gypsum to complement the diagnosis of transverse maxillary deficiency. AR, CR and CBCT were performed within the same maximum period of twenty days.

AR and CR tests were performed in compliance with the Consensus Report on Acoustic Rhinometry and Rhinomanometry of 2005,^21^ with the use of medical equipment A1/NR6 (GM Instruments^®^, Kilwinning, Scotland, United Kingdom). Nasal vasoconstrictor [oxymetazoline hydrochloride (0.5 mg/mL)] was administered in two steps: (i) two sprays of 50 μg into each nostril; (ii) a spray into each nostril after 5 min. Measurements after the use of vasoconstrictor were obtained 15–30 min after last administration.

In AR, patients were instructed to hold their breath for 3 s to perform nasal measurements. In CR, patients were instructed to close their mouth and breathe normally until four curves of inhalation and exhalation were obtained. Nasal resistance (cm^3^/s) was set at a pressure of 150 Pa.

CBCT was performed with I-Cat^®^ tomographic and panoramic imaging system (Imaging Sciences International, Hatfield. PA, USA), with the following settings: 5 mA, 120 kV, exposure time of 26.9 s, and voxel size of 0.2 mm. Osirix^®^ (Pixmeo, Geneva, Switzerland), an image-processing software dedicated to DICOM images, was used to visualize the digital three-dimensional volumetric image (3D) on axial, sagittal and coronal planes. The axial and sagittal planes have been set by the lower edges of the orbits and the hard palate, passing through orthodontic points of the anterior (ANS) and posterior nasal spines (PNS). The coronal planes were evaluated considering the orthogonal planes in relation to the sagittal and axial planes (Fig. 1).

The nasal cavity was evaluated in CBCT in two coronal sections, on the head of the inferior and middle turbinates (Fig. 1). The coronal plane passing on the head of the inferior turbinate determined the Widths 1 and 2. The coronal plane passing on the head of the middle turbinate determined the Widths 3 and 4. The coronal plane – Width 5 – was located in the middle of the mesiobuccal root canal of the mesial first upper molar. These anatomical points were established given the relation of CBCT to RA, which shows MCA1 and MCA2 values determined in the turbinate regions.

The Width 1 was measured by the horizontal line through the anterior end of the bone portion of inferior turbinates. The Width 2 was measured on the external edge of nasal cavity by the horizontal line passing through the floor of the nasal cavity. The Width 3 was measured on the internal edge of the nasal cavity by the horizontal line through the insertion of the lower turbinate. The Width 4 was measured by the horizontal line through the floor of the nasal cavity on the external edge of the palatal region of the maxillary bone. The Width 5 (Maxilla) was measured between the deepest point of the maxillary concavity bilaterally.

The data were evaluated with the use the software Statistical Package for Social Sciences (SPSS) version 22.0 (SPSS Inc., Chicago, IL, USA) and MedCalc^®^ for Windows, version 16.1 (MedCalc^®^ Software, Ostend, Belgium). For the analysis of the data, descriptive analysis (mean, median, standard deviation, minimum and maximum values) was used. The Spearman's Correlation test was used to assess the relationship between the variables of the exams. The comparison of data before and after the use of vasoconstrictor was performed by the Mann–Whitney test. Alpha value was set at 5% for all analyses.

This study was approved by the Ethics Committee for Research (#041/2011). All procedures performed in this study were in accordance with the ethical standards of the national research committee and with the 1964 Helsinki declaration and its later amendments or comparable ethical standards.

The minor's legally authorized representative was informed of the study and signed an Informed Consent Document before any research procedures started.

## Results

The descriptive analysis of the data obtained by AR, CR and CBCT examinations of the maxilla and nasal cavity is shown in Table 1.

**Table 1:** Descriptive analysis of acoustic rhinometry, computed rhinomanometry and cone-beam computed tomography of maxilla and nasal cavity in mouth breathers with transverse maxillary deficiency and comparison between the values of acoustic rhinometry and computed rhinomanometry tests, with or without the use of nasal vasoconstrictor.

Examination	Marker	Vasoconstritor	Mean	Standard deviation	Median	Minimum	Maximum
Acoustic rhinometry	Nasal cavity volume 0–5 cm (cm^3^)	With	18.77	9.10	14.18	9.29	39.44
		Without	12.02	4.81	11.16	5.75	31.18
	Nasal cavity volume 2–5 cm (cm^3^)	With	15.50	8.31	11.22	6.4	34.6
		Without	9.16	4.37	8.46	3.47	26.95
	MCA 1 (cm^2^)	With	2.71	1.84	2.13	0.98	7.38
		Without	1.49	0.97	1.24	0.74	6.06
	MCA 2 (cm^2^)	With	5.50	3.03	4.65	1.84	12.28
		Without	3.38	2.05	2.69	0.72	10.77

Computed rhinomanometry	Flow (cm^3^/s)	With	549.27	207.54	539	139	966
		Without	329.43	159.77	304	48	703
	Average inspiratory resistance (Pa/cm^3^/s)	With	1.62	1.42	1.19	0.62	6.45
		Without	3.05	2.96	2.35	0.95	17.18
	Average expiratory resistance (Pa/cm^3^/s)	With	1.52	1.15	1.07	0.70	5.17
		Without	3.81	3.82	2.70	0.98	18.02

Cone-beam computed tomography	Width 1		2.10	0.22	2.10	1.61	2.58
	Width 2		2.63	0.59	2.73	1.21	3.87
	Width 3		1.95	0.25	1.95	1.56	2.46
	Width 4		4.38	0.59	4.18	3.47	5.94
	Width 5		6	0.55	6.08	3.78	6.69

MCA1 and MCA2, minimum cross-sectional areas of the nasal cavity; Nasal vasoconstrictor spray, oxymetazoline hydrochloride 0.5 mg/mL; Widths 1 and 2, coronal section on the head of the inferior turbinate; Widths 3 and 4, coronal section on the head of the middle turbinate; Widths 5, coronal section of the maxillary width. Mann–Whitney test was used for data of acoustic rhinometry and computed rhinomanometry. All values were different with and without vasoconstrictor (*p* < 0.001). Alpha equals 0.05.

**Figure 2:**
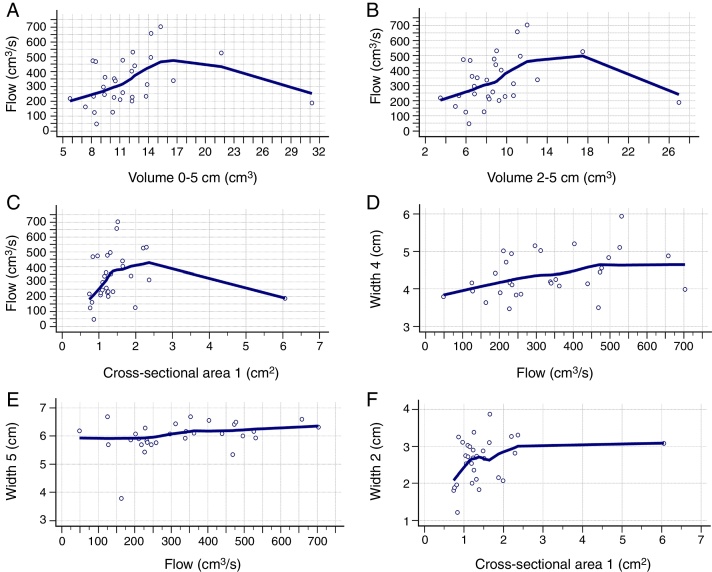
Positive correlation between the data obtained in the acoustic rhinometry, computed rhinomanometry and cone-beam computed tomography. A, Correlation between flow of computed rhinomanometry and nasal volume of 0–5 cm of acoustic rhinometry. (Rho = 0.421; 95% CI = 0.072–0.679). B, Correlation between flow of computed rhinomanometry with the nasal volume of 2–5 of acoustic rhinometry (Rho = 0.393; 95% CI = 0.038–0.660). C, Correlation between flow of computed rhinomanometry and the minimum cross-sectional area 1 of acoustic rhinometry (Rho = 0.375; 95% IC = 0.017–0.648). D, Correlation between Width 4 of cone-beam computed tomography and flow of computed rhinomanometry (Rho = 0.380; 95% CI = 0.023–0.651). E, Correlation between Width 5 of cone-beam computed tomography and flow of computed rhinomanometry. (Rho = 0.371; 95% CI = 0.013–0.645). F, Correlation between Width 3 of cone-beam computed tomography and minimum cross-sectional area 1 of the acoustic rhinometry (Rho = 0.380; 95% CI = 0.022–0.651). Statistical analysis performed by Spearman's correlation test.

**Figure 3:**
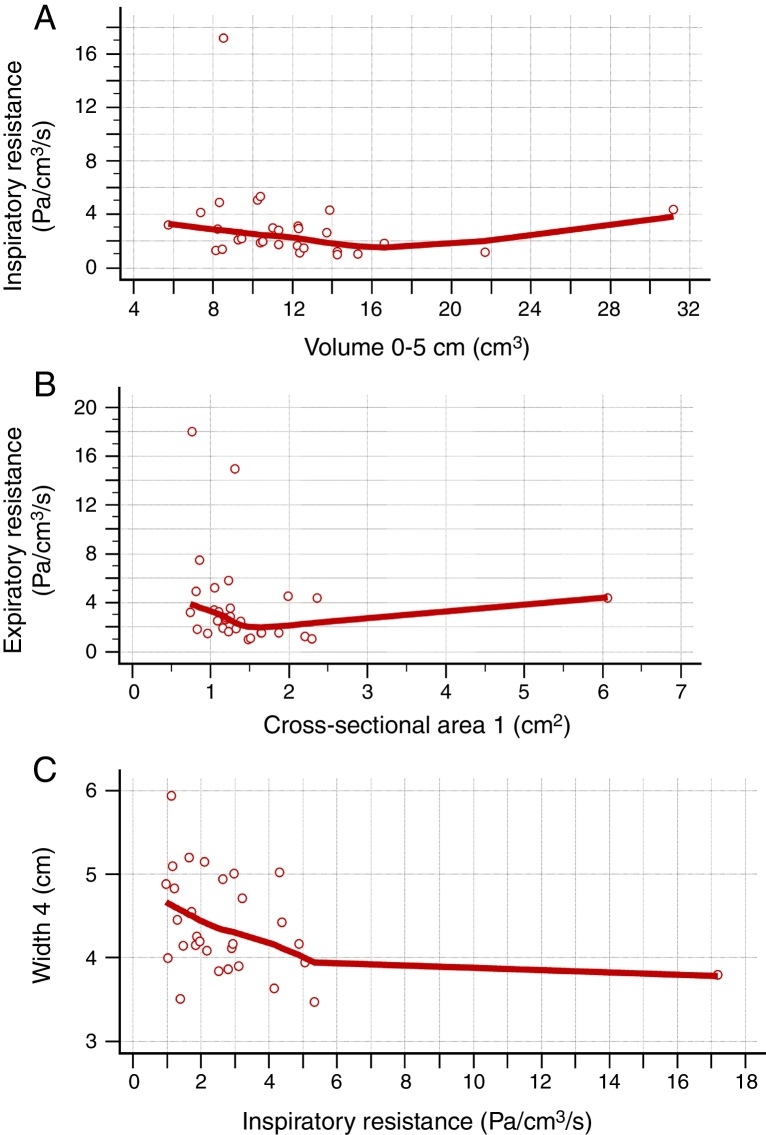
Negative correlation between the data obtained in the acoustic rhinometry, computed rhinomanometry and cone-beam computed tomography. A, Correlation between average inspiratory resistance of computed rhinomanometry and nasal volume of 0–5 cm of acoustic rhinometry (Rho = −0.382; −0.653 to −0.026). B, correlation between average expiratory resistance of computed rhinomanometry and minimum cross-sectional area 1 of acoustic rhinometry (Rho = −0.362; 95% CI = −0.639 to −0.001). C, Correlation between Width 4 of cone-beam computed tomography and average inspiratory resistance of computed rhinomanometry (Rho = −0.385; 95% CI = −0.654 to −0.029). Statistical analysis was performed by Spearman's correlation test.

Table 2 shows the correlation between AR and CR data. There was a positive correlation between nasal flow without vasoconstrictor and the nasal volume of 0–5 cm (Rho = 0.421; 95% CI = 0.072–0.679), nasal volume of 2–5 cm (Rho = 0.393; 95% CI = 0.038–0.660) and MCA1 (Rho = 0.375; 95% CI = 0.017–0.648) (Fig. 2A–C). There was a negative correlation between AIR and volume of 0–5 cm (Rho = −0.382; 95% CI = −0.653 to −0.026), as well as between AER and MCA1 (Rho = −0.362; 95% CI = −0.639 to −0.001) (Fig. 3A and B), without vasoconstrictor.

**Table 2:** Correlation between data of acoustic rhinometry and computed rhinomanometry in mouth breathers with transverse maxillary deficiency, as well as between width 5 and width 1 to 4 of cone- beam computed tomography of maxilla and nasal cavity.

Computed rhinomanometry	Acoustic rhinometry							
	Nasal cavity vol. 0–5 cm		Nasal cavity vol. 2–5 cm		Minimum cross-sectional area 1		Minimum cross-sectional area 2	
	With vasoconstrictor	Without vasoconstrictor	With vasoconstrictor	Without vasoconstrictor	With vasoconstrictor	Without vasoconstrictor	With vasoconstrictor	Without vasoconstrictor
Flow (*p*-value)	0.2926	**0.0204**	0.3648	**0.0318**	0.9265	**0.0413**	0.6927	0.0708
Rho coefficient	0.199	**0.421**	0.172	**0.393**	−0.0176	**0.375**	0.0752	0.335
95% CI	−0.174 to 0.522	**0.072 to 0.679**	−0.201 to 0.501	**0.038 to 0.660**	−0.375 to 0.345	**0.017 to 0.648**	−0.293 to 0.424	−0.029 to 0.620
AIR (*p*-value)	0.3866	**0.0370**	0.4724	0.0550	0.7649	0.0988	0.8253	0.1593
Rho coefficient	−0.164	−**0.382**	−0.136	−0.354	0.0570	−0.307	−0.0421	−0.263
95% CI	−0.495 to 0.209	−**0.653 to 0.026**	−0.473 to 0.235	−0.633 to 0.007	−0.310 to 0.409	−0.601 to 0.0598	−0.396 to 0.323	−0.570 to 0.107
AER (*p*-value)	0.1794	0.0983	0.2556	0.1607	0.5488	**0.0496**	0.6733	0.3347
Rho coefficient	−0.252	−0.307	−0.214	−0.263	−0.114	−**0.362**	−0.0803	−0.182
95% IC	−0.561 to 0.119	−0.601 to 0.059	−0.533 to 0.158	−0.569 to 0.108	−0.456 to 0.257	**−0.639 to −0.001**	−0.428 to 0.288	−0.509 to 0.190
Tomography	**Width 1**		**Width 2**		**Width 3**		**Width 4**	
Width 5 (*p*-value)	**0.020**		**0.0242**		0.0950		**0.0181**	
Rho coefficient	**0.542**		**0.411**		0.310		**0.429**	
95% CI	**0.226**–**0.755**		**0.059**–**0.672**		-0.056–0.603		**0.081**–**0.683**	

Vol., volume; 95% CI, confidence Interval of 95% for Rho coefficient; AIR, average inspiratory resistance; AER, average expiratory resistance. Statistical analysis was performed by Spearman's correlation test. Positive data are in bold type. Alpha equals 0.05.

For the CR and CBCT correlation, there was a positive correlation between flow without the use of vasoconstrictor and Width 4 (Rho = 0.380; 95% CI = 0.023–0.651) and Width 5 (Rho = 0.371; 95% CI = 0.013–0.645) (Fig. 2D and E), as well as a negative correlation between AIR and Width 4 (Rho = −0.385; 95% CI = −0.654 to −0.029) (Fig. 3C and Table 3).

**Table 3:** Correlation between data of cone-beam computed tomography and computed rhinomanometry in mouth breathers with transverse maxillary deficiency.

Cone-beam computed tomography	Computed rhinomanometry					
	Flow		AIR		AER	
	With vasoconstrictor	Without vasoconstrictor	With vasoconstrictor	Without vasoconstrictor	With vasoconstrictor	Without vasoconstrictor
Width 1 (*p*-value)	0.3312	0.7293	0.2159	0.8821	0.3874	0.6032
Rho coefficient	−0.184	0.0659	0.233	−0.0283	0.164	0.0989
95% CI	−0.510 to 0.189	−0.302 to 0.416	−0.139 to 0.547	−0.385 to 0.335	−0.209 to 0.495	−0.271 to 0.443
Width 2 (*p*-value)	0.2774	0.1790	0.2658	0.3449	0.0752	0.1077
Rho coefficient	0.205	0.252	−0.210	−0.179	−0.330	−0.300
95% CI	−0.168 to 0.526	−0.119 to 0.561	−0.530 to 0.163	−0.506 to 0.194	−0.617 to 0.035	−0.596 to 0.068
Widht 3 (*p*-value)	0.4810	0.2560	0.4847	0.6859	0.2793	0.9015
Rho coefficient	−0.134	0.2014	0.133	−0.0770	0.204	−0.0236
95% CI	−0.471 to 0.235	−0.158 to 0.533	−0.239 to 0.470	−0.425 to 0.291	−0.169 to 0.526	−0.381 to 0.340
Width 4 (*p*-value)	0.4982	**0.0384**	0.6126	**0.0357**	0.2311	0.0816
Rho coefficient	0.129	**0.380**	−0.0963	−**0.385**	−0.225	−0.323
95% CI	−0.243 to 0.467	**0.023 to 0.651**	−0.441 to 0.273	−**0.654 to** −**0.029**	−0.542 to 0.147	−0.612 to 0.042
Width 5 (*p*-value)	0.4691	**0.0433**	0.570	0.0764	0.4007	0.1424
Rho coefficient	0.137	**0.371**	−0.103	−0.328	−0.159	−0.241
95% CI	−0.235 to 0.474	**0.013 to 0.645**	−0.447 to 0.237	−0.616 to 0.036	−0.491 to 0.213	−0.578 to 0.095

95% CI, confidence interval of 95% for Rho coefficient; AIR, average inspiratory resistance; AER, average expiratory resistance. Statistical analysis was performed by Spearman's correlation test. Positive data are in bold type. Alpha equals 0.05.

For the AR and CBCT correlation, there was a positive correlation between MCA1 evaluated without administration of vasoconstrictor and Width 2 (Rho = 0.380; 95% CI = 0.022–0.651) (Fig. 2F); Width 4 and nasal volumes of 0–5 cm evaluated without administration of vasoconstrictor (Rho = 0.376; 95% CI = 0.018–0.648), nasal volumes 2–5 cm evaluated without administration of vasoconstrictor (Rho = 0.376; 95% CI = 0.018 to 0.648), MCA1 evaluated without administration of vasoconstrictor (Rho = 0.410; 95% CI = 0.058–0.671); and Width 4 and MCA1 after administration of vasoconstrictor (Rho = 0.426; 95% CI = 0.077–0.682) (Table 4).

**Table 4:** Correlation between data of cone-beam computed tomography and acoustic rhinometry in mouth breathers with transverse maxillary deficiency.

Cone-beam computed tomography	Acoustic rhinometry							
	Nasal cavity vol. 0–5 cm		Nasal cavity vol. 2–5 cm		Minimum cross-sectional area 1		Minimum cross-sectional area 2	
	With vasoconstrictor	Without vasoconstrictor	With vasoconstrictor	Without vasoconstrictor	With vasoconstrictor	Without vasoconstrictor	With vasoconstrictor	Without vasoconstrictor
Width 1 (*p*-value)	0.9534	0.2050	0.8830	0.2442	0.4223	0.2932	0.5288	0.9529
Rho coefficient	−0.011	0.238	0.028	0.219	0.152	0.198	0.120	0.0113
95% CI	−0.370 to 0.351	−0.134 to 0.551	−0.336 to 0.384	−0.153 to 0.537	−0.220 to 0.486	−0.174 to 0.521	−0.251 to 0.460	−0.350 to 0.370
Width 2 (*p*-value)	0.3775	0.2742	0.4242	0.3281	0.3221	**0.0385**	0.4150	0.5556
Rho coefficient	0.167	0.206	0.152	0.185	0.187	**0.380**	0.154	0.112
95% CI	−0.206 to 0.497	−0.166 to 0.527	−0.221 to 0.485	−0.188 to 0.511	−0.186 to 0.513	**0.022 to 0.651**	−0.218 to 0.488	−0.259 to 0.454
Width 3 (*p*-value)	0.3387	0.6353	0.3832	0.7242	0.4977	0.5196	0.6780	0.9246
Rho coefficient	−0.181	0.090	−0.165	0.067	−0.129	0.122	−0.0791	0.0180
95% CI	−0.508 to 0.192	−0.279 to 0.436	−0.496 to 0.207	−0.300 to 0.417	−0.467 to 0.243	−0.249 to 0.462	−0.427 to 0.289	−0.344 to 0.376
Width 4 (*p*-value)	0.3622	**0.0408**^a^	0.444	**0.0408**^a^	**0.0190**	**0.0245**	0.2770	0.5727
Rho coefficient	0.172	**0.376**	0.145	**0.376**	**0.426**	**0.410**	0.227	0.107
95% CI	−0.200 to 0.502	**0.018 to 0.648**	−0.227 to 0.480	**0.018 to 0.648**	**0.077 to 0.682**	**0.058 to 0.671**	−0.145 to 0.543	−0.263 to 0.450
Width 5 (*p*-value)	0.9311	0.0955	0.9916	0.1152	0.9441	0.1543	0.9246	0.4411
Rho coefficient	−0.017	0.310	0.002	0.294	0.0134	0.267	0.0180	0.146
95% CI	−0.375 to 0.346	−0.057 to 0.603	−0.359 to 0.362	−0.075 to 0.591	−0.349 to 0.372	−0.104 to 0.572	−0.344 to 0.376	−0.226 to 0.481

Vol., volume; 95% CI, confidence interval of 95% for Rho coefficient. Statistical analysis was performed by Spearman's correlation test. Positive data are in bold type. Alpha equals 0.05.

a The same Rho coefficient and 95% CI were observed.

Width 5 showed positive correlation between Width 1 (Rho = 0.542; 95% CI = 0.226–0.755), Width 2 (Rho = 0.411; 95% CI = 0.059–0.672), and Width 4 (Rho = 0.429; 95% CI = 0.081–0.683) (Table 2, data are not presented graphically).

## Discussion

The relationship of nasal respiratory function with the development of dentofacial alterations is controversial. Mouth breathing was related to: (i) the development of posterior crossbite and increased dental overjet in patients with allergic rhinitis^1^, ^22^; (ii) highest prevalence of hard, narrow, and high-arched palate^3^, ^23^; (iii) hypertrophic adenoids with markedly convex facial profile^4^; (iv) severe mouth breathing with changes in the nasolabial profile.^24^ However, in other reports, there was no correlation between mouth breathing and facial pattern or malocclusions.^7^, ^8^ Genetic factors are likely to contribute to the presence of deficiency; therefore, further studies should be carried out.

In this study, the narrowing of the maxillary bone (Width 5) and the nasal base width (Width 4) showed a positive correlation with the nasal airflow. A negative correlation between Width 4 and AIR was also observed. There was a positive correlation between maxillary width and the nasal width in transverse maxillary deficiency. Patients with more severe deficiency, with lower maxillary width, showed lower nasal width and airflow values. Nasal Width 3 was the only one without a positive correlation with the maxilla width, probably because it was measured in the middle third of the nostril. This supports the theory that mouth breathers have impaired nasal breathing due to the presence of transverse maxillary deficiency and narrower nasal base.^1^, ^2^, ^3^

A positive correlation was identified between nasal airflow without the use of vasoconstrictor and the nasal volume of 0–5 cm, nasal volume of 2–5 cm, and MCA1. In regions with higher nasal volume, greater nasal airflow could be observed. A negative correlation was observed between the nasal volume of 0–5 cm and AIR. Patients with higher nasal volume showed less AIR. AR and CR methods are widely reported in the literature^25^, ^26^ and demonstrated correlation between each other.

Orthodontic procedures, such as rapid maxillary expansion, expand the maxillary bone, increase the width of the base of the nasal bone and can improve nasal breathing.^14^, ^15^, ^16^ However, nasal breathing is also influenced by the condition of the nasal mucosa. In case of hypertrophy of the nasal mucosa, airflow impairment will be observed. In this study, AR and CR examinations were carried out without the use of vasoconstrictor in order to evaluate patients in their natural state of health, and with the use of vasoconstrictor to reduce congestion of the nasal mucosa. All correlations were observed without the use of vasoconstrictor.

CBCT allows three-dimensional evaluation of the bone structures, with less radiation exposure than conventional CT, and with better quality of radiographic imaging than teleradiography. For this reason, CBCT is widely used for clinical purposes and in researches.^14^, ^16^ The nasal widths in coronal sections in the inferior and middle turbinate regions were compared to their respective narrowest regions, in the same anatomical regions, determined in AR (MCA1 and MCA2). There were positive correlations between MCA1 evaluated without vasoconstrictor and Width 2, as well as, Width 4 and nasal volumes of 0–5 cm evaluated without vasoconstrictor, Width 4 and nasal volumes of 2–5 cm evaluated without vasoconstrictor, Width 4 and MCA1 evaluated after administration of vasoconstrictor. The larger the area in the anatomical narrowing, the greater the nasal width.

In our data, we observed correlation between the variables of the exams with weak and moderate strength. This fact can be associated with the small sample size.

The existence of correlations among RA, CR and CBCT emphasizes the importance of a team of orthodontists, pediatricians and otorhinolaryngologists for the interdisciplinary evaluation and treatment of patients with mouth breathing.

Limitations to this study included: (i) small sample size; (ii) non-inclusion of healthy controls for correlation between the tests; (iii) non-inclusion of controls with deficiency and nasal breathers. Controls were not included due to the need of CBCT examinations. In the future, comprehensive studies should be carried out with larger sample sizes and include comparisons between the groups mentioned in the limitations to this study, as well as the results obtained from the long-term treatment of transverse maxillary deficiency with maxillary expansion. Therefore, the results of this study have a special relevance for future research challenges.

## Conclusion

Correlation was observed between nasal and maxillary widths (CBCT) and the nasal respiratory function (AR and CR) in mouth breathers with transverse maxillary deficiency.

## Funding

This work was supported by Fundação de Amparo à Pesquisa do Estado de São Paulo (FAPESP) [grant number 2012/03519-4].

## Conflicts of interest

The authors declare no conflicts of interest.
